# Nanomedicines based on nanoscale metal-organic frameworks for cancer immunotherapy

**DOI:** 10.1038/s41401-020-0414-6

**Published:** 2020-04-30

**Authors:** Xiao-fang Zhong, Xun Sun

**Affiliations:** Key Laboratory of Drug-Targeting and Drug Delivery System of the Education Ministry, Sichuan Engineering Laboratory for Plant-Sourced Drug and Sichuan Research Center for Drug Precision Industrial Technology, West China School of Pharmacy, Sichuan University, Chengdu, 610064 China

**Keywords:** cancer immunotherapy, nanoscale metal-organic frameworks (nMOFs), cancer vaccine, in situ vaccination, immunomodulators, immune response

## Abstract

Cancer immunotherapy, with an aim to enhance host immune responses, has been recognized as a promising therapeutic treatment for cancer. A diversity of immunomodulatory agents, including tumor-associated antigens, adjuvants, cytokines and immunomodulators, has been explored for their ability to induce a cascading adaptive immune response. Nanoscale metal-organic frameworks (nMOFs), a class of crystalline-shaped nanomaterials formed by the self-assembly of organic ligands and metal nodes, are attractive for cancer immunotherapy because they feature tunable pore size, high surface area and loading capacity, and intrinsic biodegradability. In this review we summarize recent progress in the development of nMOFs for cancer immunotherapy, including cancer vaccine delivery and combination of in situ vaccination with immunomodulators to reverse immune suppression. Current challenges and future perspectives for rational design of nMOF-based cancer immunotherapy are also discussed.

## Introduction

Over the past several decades, immunotherapy has gained significant attention as a new powerful treatment against cancer. The major goal of cancer immunotherapy is to awaken and strengthen the immune system [1–3]. Various cancer immunotherapy strategies have been investigated, such as cancer vaccines and immune checkpoint therapy [4, 5]. For example, several vaccines have been designed to target “universal” tumor antigens shared by many patients, but they have shown only modest clinical successes, in part because of weak immunogenicity [6]. Neoantigens can bind to T cell receptors (TCRs) with higher affinity than tumor-associated antigens, and they can induce more robust T cell responses [7]. However, patient-specific neoantigens have yet to be identified [8].

Rather than generating the cancer vaccine in vitro from isolated neoantigens, some researchers have attempted to generate vaccines in vivo which has been termed in situ vaccination [9]. This approach avoids the need to identify and isolate neoantigens, and it can exploit the entire repertoire of antigens expressed by tumors in a given patient, allowing the development of personalized vaccines. Ideally, an in situ vaccine should induce local cancer cell death, which facilitates the release of tumor antigens, and it should enhance antigen uptake and activation of antigen-presenting cells (APCs) to elicit antitumor T cell responses [10]. These processes can be achieved through photodynamic therapies, radiotherapies and certain chemotherapies, such as anthracyclines and oxaliplatin [11].

Nanotechnology may offer unique possibilities for cancer immunotherapy because nanoparticles can serve as passive vehicles for transporting immunostimulatory agents such as antigens and adjuvants, protecting these agents from degradation and delaying their removal from the body [12, 13]. In addition, nanomedicines that properly integrate nanotechnology can selectively target lymph nodes, immune cells, and tumor sites to improve therapeutic efficacy [14, 15].

Accordingly, various types of nanocarriers have been developed, such as liposomes [16, 17], micelles [18, 19], mesoporous silica nanoparticles [20] and nanosized metal-organic frameworks (nMOFs). nMOFs, which are hybrid porous materials built from metal ions or clusters and organic bridging ligands, have gained much popularity over the past two decades [21]. nMOFs have been designed using diverse synthetic strategies and applied to gas separation, catalysis, and energy storage and, more recently, drug delivery [22–25]. nMOFs intrinsically possess large surface areas, highly ordered porosities, and well-defined structures, which endow these materials with the capability of loading and releasing different cargos, especially therapeutic agents. The most often studied nMOFs are Materials of the Institute Lavoisier (MIL), zeolitic imidazolate frameworks (ZIFs), porous coordination networks (PCNs) and University of Oslo (UIO) nanoparticles. For example, MIL-53(Fe), composed of terephthalate anions and Fe(III) octahedra, was used to adsorb ibuprofen with a loading capacity of 20% (wt) [26]. A complete release of ibuprofen took approximately 21 days, which was proven to have zero-order kinetics. ZIF-8 has a porous polymeric network structure made up of zinc metal centers tetrahedrally coordinated to 2-methylimidazole ligands, giving a sodalite topology with a surface of ~1600 m^2^/g and a six-ring pore aperture of 3.4 Å.

nMOFs possess the following unique advantages that enable them to perform as promising platforms for drug delivery and cancer immunotherapy. First, their versatile structures provide nMOFs with diverse morphologies, compositions, sizes and chemical properties, which endow them with multifunctionalities such as lymph node-targeting ability and the ability to codeliver antigens and immunomodulators. Second, large surface areas and high porosities endow nMOFs with much higher antigen/adjuvant/cytokine loading capacity than liposomes or micelles. Third, labile metal-ligand bonds ensure nMOFs are degraded at specific sites such as endosomes/lysosomes or tumor cells, which provides relatively controlled drug release when compared with that of mesoporous silica nanoparticles [27–29]. The literature describes at least four ways in which drugs can be loaded into nMOFs: (i) physical adsorption onto the outer crystal surface, (ii) covalent conjugation to the framework, (iii) noncovalent binding within sufficiently large pores of the framework, and (iv) coprecipitation with the framework during nMOF self-assembly under physiological conditions.

These four methods have proven effective for loading nMOFs with antigens, adjuvants and immunomodulators for cancer immunotherapy [30, 31]. The large surface area of nMOFs means they can accommodate large amounts of drugs or other cargo, and their tunable structure means they can easily be functionalized or modified for specific purposes, such as targeting immune cells. The relatively liable bonds between the metal and ligands facilitate the release of the cargo at the target site.

**Fig. 1:**
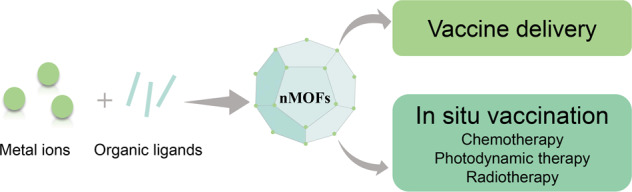
Schematic illustration of the application of nanosized metal-organic frameworks (nMOFs) in cancer immunotherapy. These applications include vaccine delivery and in situ vaccination.

This review covers recent progress in using nMOFs for cancer immunotherapy. First, we illustrate the benefit of using nMOFs for the delivery of cancer vaccines. Second, we provide examples of nMOFs that serve as in situ cancer vaccines and have been simultaneously combined with immunomodulators (Fig. 1). We conclude by briefly discussing challenges and perspectives in the application of nMOFs in cancer immunotherapy.

## Cancer vaccine delivery based on nMOFs

nMOFs are compositionally and structurally diverse, allowing the facile synthesis of nMOFs with suitable shapes, sizes and chemical properties. Given their characteristic high loading capacity, nMOFs are exceedingly attractive as cancer vaccine vehicles to codeliver antigens and adjuvants to APCs or lymph nodes.

### Codelivery of antigens and adjuvants to enhance the uptake of APCs using nMOFs

Using antigens on their own as vaccines usually elicits weak immune responses because the antigens show low immunogenicity and/or off-target effects [32]. Immune responses are usually stronger when the antigens are loaded into nanoparticles or conjugated to them. The high loading capacity of nMOFs means that they can be engineered as cancer vaccine platforms to codeliver antigens and adjuvants to enhance their detection by APCs, which process them, display them in complex with the major histocompatibility complex (MHC) molecules on their surface, and thereby activate antigen-specific T cells [33]. For example, Qu and coworkers loaded ovalbumin (OVA) into ZIF-8, and cytosine-phosphate-guanine oligodeoxynucleotide (CpG ODN) was adsorbed into 200-nm particles as an adjuvant. The nMOFs were efficiently internalized by RAW264.7 cells, and they elicited a potent memory immune response [34]. Zhang et al. loaded OVA into complexes of Eu^2+^ and guanine monophosphate (GMP) in one-pot coprecipitation, and CpG was allowed to adsorb via Watson–Crick base pairing. Eu^2+^ and GMP acted as nMOF coordinating partners. The nMOFs were taken up by RAW264.7 cells to a greater extent than OVA alone or a simple mixture of free OVA and CpG. At pH values below 5.0, the metal-ligand bonds broke, destroying the nMOFs and releasing OVA. The nMOFs triggered higher secretion of the Th1-polarizing cytokine TNF-α than OVA alone or OVA/CpG [35].

Cancer vaccines can be prophylactic (intended to prevent future infection) or therapeutic (intended to treat existing infection) [36]. Prophylactic cancer vaccines have proven effective in a few cases, such as in cancers related to human papillomavirus infection or chronic infection with hepatitis B virus [37, 38]. In contrast, developing therapeutic cancer vaccines has proven much more challenging. Therapeutic vaccines should elicit systemic immunity, especially cellular-mediated immunity. They should trigger the expansion and differentiation of antigen-specific CD8^+^ T cells into cytotoxic T lymphocytes (CTLs), which kill cancer cells and generate long-living CD8^+^ memory T cells. Vaccines can trigger these effects by delivering antigen to APCs, among which dendritic cells (DCs) are particularly effective at stimulating T cells [39]. More importantly, CD8a^+^ DCs and/or CD103^+^ DCs can specifically present exogenous antigens on MHC-I molecules and thereby prime CTLs, a process called “cross-presentation” [40, 41]. Xue and coworkers [42] developed MIL-101-Fe-NH_2_ to codeliver OVA and CpG in mice, which led to much higher uptake by DCs and immune responses by CTLs and other immune cells than the mixture of OVA and CpG.

### Vaccine vehicles for lymph node targeting

Lymph nodes contain many phagocytically active DCs, including lymph node-resident CD8^+^ DCs, and they are therefore the main sites of immune activation and surveillance [41, 43, 44]. Studies have confirmed that delivering antigens to lymph nodes can improve antigen-specific adaptive immune responses [45, 46]. The size of nanovesicles appears to be the most important factor for lymph node targeting: particles smaller than 100 nm are more likely to drain into lymph nodes [47]. nMOFs are compositionally and structurally diverse, allowing the facile synthesis of nMOFs with suitable shapes, sizes and chemical properties for lymph node targeting.

**Fig. 2:**
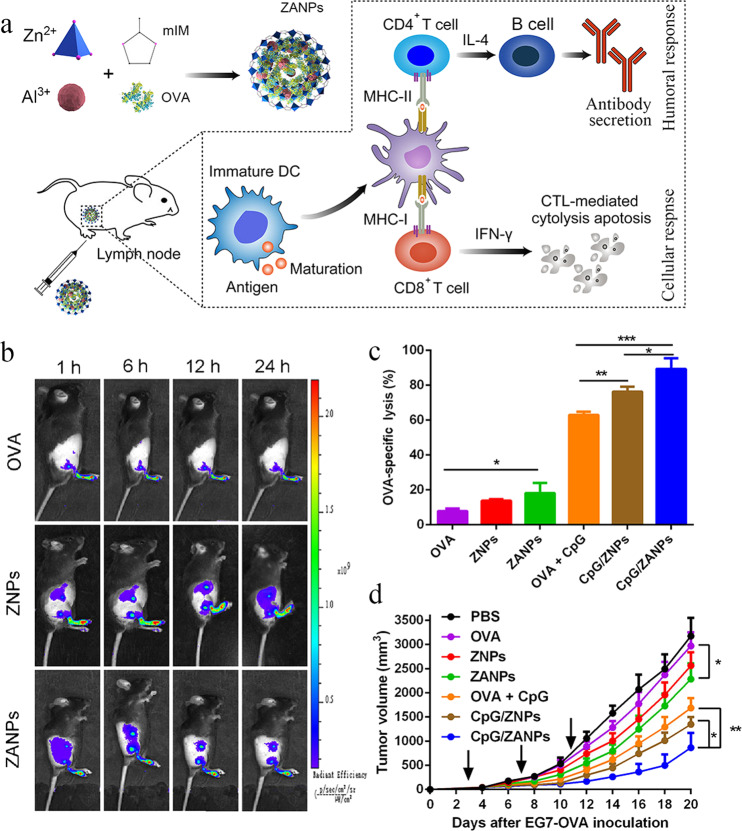
Lymph node-targeting nMOFs as an efficient cancer vaccine delivery platform. **a** Schematic illustration of Zn^2+^-based nMOFs containing aluminum adjuvant and OVA (ZANPs), and how they evoke efficient humoral and cellular immune responses. **b** In vivo near-infrared fluorescence imaging of different formulations at 1, 6, 12 or 24 h after administration in the footpad. **c** OVA-specific CTL response elicited by different formulations, based on flow cytometry of CFSE labeling. **d** Tumor volume from mice challenged with EG7-OVA (EL-4 thymoma tumor cells transfected with the OVA gene) cells. *P < 0.05, **P < 0.01, ***P < 0.001. CTL cytotoxic T lymphocyte, DCs dendritic cells, mIM imidazole, OVA ovalbumin, ZNP Zn^2+^-based nMOFs containing OVA but no adjuvant, ZANPs Zn^2+^-based nMOFs containing aluminum adjuvant and OVA. This figure was adapted with permission from ref. [48]. Copyright 2019 Elsevier B.V.

Taking advantage of biomineralization, Sun et al. loaded aluminum adjuvant integrated-ZIF-8 with OVA, and they coated the nanoparticles with CpG via electrostatic interaction to boost Th1-type immunity. The resulting 80-nm nanoparticles (CpG/ZANPs) efficiently codelivered OVA and CpG to lymph nodes, where the same DCs phagocytosed both antigen and adjuvant (Fig. 2a, b). Within the acidic lysosomes, the imidazole in ZIF-8 became protonated, disrupting its interaction with zinc ions, thereby allowing OVA to escape from lysosomes into the cytoplasm and be cross-presented by DCs. These nanoparticles induced a greater proportion of CD8^+^ T cells secreting IFN-γ^+^ as well as higher IgG2a antibody secretion than the mixture of OVA and CpG. Consistent with the enhanced antigen-specific CTL response, CpG/ZANPs significantly inhibited tumor growth and prolonged survival in mice bearing EG7-OVA cells (EL-4 thymoma tumor cells transfected with the OVA gene) (Fig. 2c, d).

Taken together, these results show that the nMOF-based vaccine delivery system offers the following advantages: (i) nMOFs have an extremely high antigen loading capacity; (ii) nMOFs can increase antigen immunogenicity and uptake by APCs; (iii) the nMOFs degraded in the acidic endo/lysosomes enable release of the encapsulated antigens into the cytoplasm, leading to enhanced antigen cross-presentation ability; and (iv) the compatibility of nMOFs with various metal ions and organic linkers allows tailoring of the system to different target tissues, such as lymph nodes.

## In situ cancer vaccination with nMOFs for combinatorial therapy

To be effective, cancer vaccines should elicit immune responses to antigens expressed by tumors. To achieve this goal, the past several decades in cancer vaccination have been characterized by considerable effort into the discovery of tumor-associated antigens. However, these tumor-associated antigen-based vaccines have proven ineffective in animal models and clinical trials. An alternative is to design vaccines based on neoantigens, which are solely expressed in cancer and not normal cells. Therefore, neoantigen-based cancer vaccines may be more specific, more effective and less toxic than vaccines targeting tumor-associated antigens. However, neoantigens are quite difficult to isolate, and the best approach for predicting them is unknown [49, 50].

Instead, researchers have begun to explore vaccines formed in situ based on the full array of the patient’s tumor antigens. The concept of in situ vaccination involves any approach that exploits antigens available at a tumor site to induce a tumor antigen-specific adaptive immune response. Tumor cells are killed by chemotherapy, radiotherapy or photodynamic therapy to release antigens, which recruit APCs to the tumor, and these APCs take up and process the antigens while being trafficked to tumor-draining lymph nodes. There, the APCs activate CTLs, which invade tumors and destroy antigen-expressing tumor cells [49, 51]. Such an approach uses antigens of patients themselves, thereby eliminating the need to previously identify and isolate the neoantigens [52–54]. Moreover, the antitumor response can be strengthened by simultaneously applying other immunotherapies [55, 56]. Recently, nMOFs have been investigated as a possible vehicle for effective in situ cancer vaccination, including in concert with chemotherapy, photodynamic therapy and radiotherapy.

### Chemical drug-mediated in situ vaccination using nMOFs

It was reported that chemotherapy could be used for in situ vaccination to promote antitumor immune responses. Certain chemotherapeutics, such as the anthracyclines doxorubicin, idarubicin and oxaliplatin, can induce so-called immunogenic cell death, which releases calreticulin, ATP and high-mobility group box protein 1 (HMGB1) into the extracellular milieu [57]. These damage-associated molecular patterns (DAMPs) induce death and apoptosis of tumor cells as well as their engulfment by DCs, increasing the number of T lymphocytes and the ratio of CD8^+^ CTLs to FOXP3^+^ regulatory T cells [58–60].

Lin and coworkers encapsulated doxorubicin and catalase into ZIF-8 nanoparticles, which were then coated with cell membrane from murine melanoma cells. This tumor cell “coating” allowed the nMOFs to escape immune surveillance and accumulate in tumor tissues. Since doxorubicin triggers immunogenic cell death, these coated nMOFs increased the number of antigen-specific CD3^+^CD8^+^ tumor-infiltrating T cells. At the same time, the nMOFs downregulated the expression of hypoxia-inducible factor-1α, reducing the risk of drug resistance and immune escape [61]. As PD-L1 expressed by cancer cells can actually inhibit antigen-specific CTLs from recognizing and killing tumor cells and even drive CTLs into apoptosis, these coated nMOFs were combined with in situ vaccination with a monoclonal antibody against PD-1. This approach increased the number of infiltrating CD8^+^ T cells and the production of interleukin-12 and tumor necrosis factor-α, inhibiting the growth and metastasis of murine melanoma tumor cells in mice [62].

### Photodynamic therapy-mediated in situ vaccination using nMOFs

Photodynamic therapy involves exposing a photosensitizer to visible or near-infrared light to generate reactive oxygen species (ROS), particularly singlet oxygen, which cause necrosis or apoptosis of tumor cells. The resulting release of DAMPs activates macrophages and DCs, which migrate to lymph nodes, where they activate humoral and cell-mediated immune responses [63, 64]. In parallel, photodynamic therapy triggers the release of inflammatory mediators and cytokines into the tumor, stimulating an adaptive immune response [65]. A powerful demonstration of this type of in situ vaccination is found in mice: mice vaccinated with lysates of photodynamically treated (Photofrin as photosensitizer) tumor cells resisted challenge with the same tumor type. Subsequent studies have revealed that other photosensitizers can also be used for generating in situ vaccines [66, 67]. This effective response seems to require activation of CD8^+^ T cells [68].

**Fig. 3:**
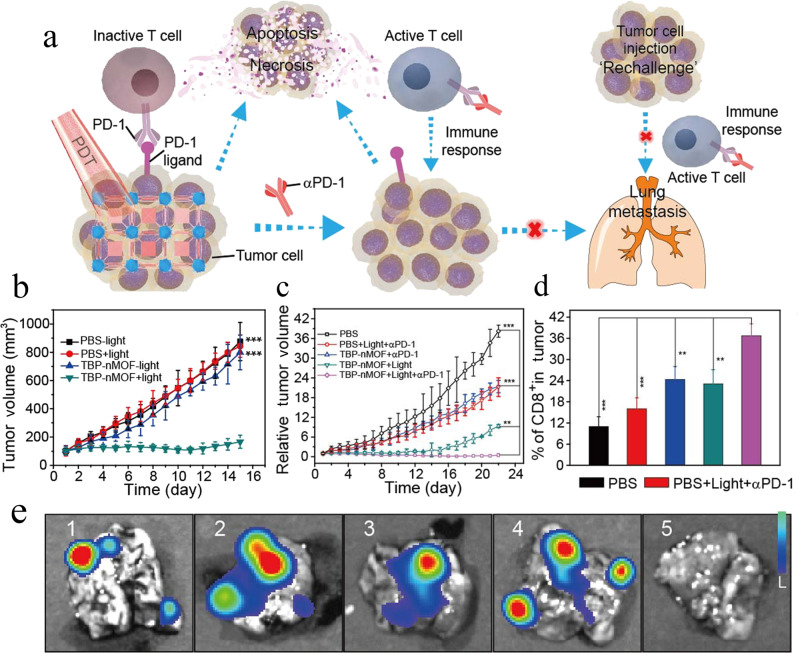
A Zr_6_-connected nMOF integrating a benzoporphyrin-based photosensitizer (TBP-nMOF) for photodynamic treatment (PDT) and combination immunotherapy. **a** Proposed mechanism of antitumor immune responses induced by TBP-nMOF, and the synergy between photodynamic therapy and anti-PD-1 antibody to inhibit tumor metastasis. **b**, **c** Tumor volume changes after **b** photodynamic therapy (light) alone or **c** combined PDT with antibody against PD-1 (α-PD-1). **d** Percentage of CD8^+^ T cells that infiltrated the tumors after the treatments in **c**. **e** Bioluminescence images of the lung from mice bearing luciferase-expressing primary 4T1 tumors on the right back of hind leg region after various treatment; 1: PBS; 2: PBS + light + α-PD-1; 3: TBP-nMOF + α-PD-1; 4: TBP-nMOF + light; 5: TBP-nMOF + light + α-PD-1. **P < 0.01, ***P < 0.001. This figure was adapted with permission from ref. [70]. Copyright 2018 American Chemical Society.

Although various photosensitizers can be effective for in situ vaccination mediated by photodynamic therapy, most of them show low aqueous solubility or poor photostability. To address these limitations, MIL-100 (Fe) was used to encapsulate different kinds of photosensitizers, notably 2-((4′-(2,2-*bis*(4-methoxyphenyl)-1-phenylvinyl)-[1,1′-biphenyl]- 4-yl)(phenyl)methylene)malononitrile (TPEDC), (*E*)-2-(4-(4-(2,2-*bis*(4-methoxyphenyl)-1-phenylvinyl)styryl)-3-cyano-5,5-dimethylfuran-2(5*H*)-ylidene)malononitrile (TPETCF) and chlorin e6 (Ce6) [69]. As they contain iron(III), MIL-100 (Fe) nanoparticles could catalyze the decomposition of H_2_O_2_ into O_2_, which relieves tumor hypoxia and potentiates photodynamic therapy. Zhang et al. [70] integrated a tetra(*p*-benzoato)porphyrin-based photosensitizer into a Zr_6_-connected nMOF measuring 100 nm (Fig. 3a). The TBP-nMOF showed stronger infrared luminescence and redshifted absorption than traditional porphyrin-based MOFs, and it generated much higher amounts of singlet oxygen even under low oxygen concentrations. The benzoporphyrin-containing nMOF not only induced the apoptosis of 4T1 murine breast cancer cells but also stimulated a strong increase in the number of tumor-infiltrating CD8^+^ and CD4^+^ T cells, and combining it with an antibody against PD-1 led to complete tumor elimination without recurrence in 4T1-bearing mice (Fig. 3b–d). The combination of photodynamic therapy and an antibody against PD-1 synergistically recruited tumor-infiltrating lymphocytes and inhibited metastasis of 4T1 tumors to the lungs (Fig. 3e).

Lin and his colleagues integrated benzoporphyrin with Fe_3_O as the metal clusters to construct Fe_3_O-based nMOFs measuring 100 nm. This nMOF was taken up efficiently by CT26 cells, and it decreased H_2_O_2_ levels through a Fenton-like reaction and downregulated hypoxia-inducible factor-1α, showing that it could alleviate hypoxia in tumor tissues. After photodynamic treatment, this Fe_3_O-based nMOF caused tumor cell death effectively through immunogenic cell death, which was investigated by detecting cell surface exposure of calreticulin. Combining the nMOF with photodynamic therapy led to tumor regression, and the further addition of anti-PD-L1 antibody significantly expanded CD4^+^ and CD8^+^ CTL populations. This reversed PD-L1-mediated immunosuppression, causing complete regression of primary tumors and 90% regression of metastatic tumors in mice [71].

Through solvothermal reactions between benzoporphyrin and WCl_6_, benzoporphyrin was incorporated into a W-based nMOF [72]. Anionic CpG as an adjuvant was adsorbed on the surface of the cationic nMOF. Photodynamic therapy triggered the release of antigens from tumors, while CpG promoted antigen internalization and presentation by DCs, which were reflected in the upregulation of MHC-II and costimulatory CD86 molecules. The resulting immune response was strong, as reflected by increases in IFN-α and IL-6. Further addition of an antibody against PD-L1 led to tumor regression in a mouse model of bilateral breast cancer.

### Radiotherapy-mediated in situ vaccination using nMOFs

Radiotherapy destroys tumor tissue using ionizing radiation, which generates damaging hydroxyl radicals in an X-ray dose-dependent manner [73]. Immunogenic cell death induces tumor-associated antigen release and translocation of calreticulin to the tumor cell membrane, which acts as an “eat me” signal to DCs, ultimately activating intratumoral DCs that upregulate the costimulatory molecules CD86 and CD70. This makes tumor antigens available for cross-presentation on MHC-I molecules, where they can prime tumor-specific T cells [74, 75].

Radiotherapy promotes the delivery of not only tumor-associated antigens but also tumor DNA to DCs, which activates type I IFN production via the “stimulator of interferon genes” (STING) pathway [76, 77]. At the same time, radiotherapy induces the production of chemokines, which recruit effector T cells to tumors. Emerging evidence indicates that radiotherapy can convert tumors into an in situ vaccine, and combining radiotherapy with immunotherapy will stimulate a systemic immune response to reject tumor cells [78].

**Fig. 4:**
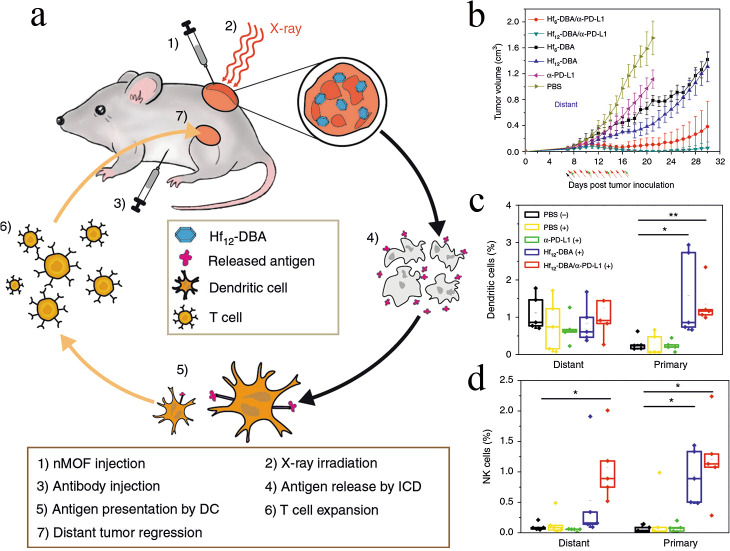
An Hf-based nMOF serve as radioenhancer, integrated low-dose radiation for immune response stimulation. **a** Abscopal effect of nMOF-enhanced radiotherapy: Hf-based nMOF triggers immunogenic cell death, which releases tumor antigen serve as in situ vaccine, while anti-PD-1 antibody reverses the immunosuppressive tumor microenvironment, enhancing T cell expansion and tumor infiltration. DC, dendritic cells. **b** Growth of distant tumors after mice were injected different formulations bilaterally on the right back of hind leg region with CT26 colorectal tumor cells. **c**, **d** Primary and distant tumors were collected and analyzed for content of tumor-infiltrating DCs natural killer (NK) cells. This figure was adapted with permission from ref. [80]. Copyright 2018 Springer Nature.

As lower doses of radiation often fail to elicit sufficiently strong immune responses but higher doses can injure off-target tissues, researchers have explored nMOFs as radioenhancers to combine with low-dose radiation. The released antigens serve as in situ individualized tumor vaccines to synergize with immune checkpoint inhibitors to inhibit tumor growth [79]. For example, nMOFs were studied as an in situ vaccine in combination with immune checkpoint inhibitors with a low radiation dose to achieve systemic rejection of colorectal tumors in mouse models [80]. A Hf-based nMOF was synthesized through coordination between Hf_12_O_8_(OH)_14_ and 2,5-di(*p*-benzoato)aniline (Fig. 4a). This nMOF led to much higher production of hydroxyl radicals than HfO_2_, indicating that it may serve as a radioenhancer since hydroxyl radicals are the major cytotoxic radical species induced by ionizing radiation. The Hf nMOF-mediated radiation treatment caused immunogenic cell death, reflected in the expression of calreticulin and HMGB1, and substantially improved the ability of Hf to shrink CT26 colorectal xenografts in mice (Fig. 4b). Combining the Hf nMOF-mediated radiotherapy with an anti-PD-L1 antibody not only inhibited local irradiated tumors but also shrank distant, nontreated tumors (Fig. 4b). Flow cytometry analysis of excised tumors showed increased numbers of IFN-γ-producing CD4^+^ and CD8^+^ T cells. Primary tumors contained higher percentages of DCs and natural killer cells than did metastases (Fig. 4c, d), suggesting that both cell types were recruited to tumors after radiation.

Similarly, Lin and his coworkers reported an nMOF constructed from a chlorin derivative, 5,10,15,20-tetra(*p*-benzoato)chlorin, which could absorb X-ray photons and induce the emission of photoelectrons via the photoelectric effect. When the pores of this nMOF were loaded with an inhibitor of indoleamine 2,3-dioxygenase and the nMOF was administered together with low-dose radiotherapy, the growth of primary and metastatic CT26 tumors was slowed in mice. Dioxygenase is overexpressed in tumor cells, where it degrades tryptophan and produces kynurenine, leading to T cell anergy and apoptosis, helping tumors evade the immune system [81]. This synergistic treatment increased the numbers of tumor-infiltrating CD45^+^ DCs and macrophages [82].

These various examples illustrate how combining appropriate nMOFs with radiotherapy can make them efficient in situ vaccines, which can synergize with immunomodulators to amplify systemic antitumor immunity.

## Summary and perspectives

Immunotherapies based on nMOFs have shown strong promise for cancer treatment. These therapies fundamentally activate an immune response through two mechanisms. One is to directly deliver tumor-associated antigens and Toll-like receptor agonists to APCs such as DCs and macrophages. This promotes APC maturation, antigen cross-presentation and T cell priming. The other mechanism is to deliver drugs or apply light or X-ray radiation to allow in situ cancer vaccination. Both mechanisms promote antigen presentation and stimulate T cell proliferation and differentiation into CTLs as well as secretion of IFN-γ, TNF-α and other cytokines.

Although nMOF-based immunotherapy has progressed substantially in the past several years, many chemical and immunological challenges stand in the way of clinical application. Undoubtedly, the most important challenges are the risk of undesired cytotoxic and genotoxic effects. Indeed, cytotoxicity was observed to strongly depend on the nMOF composition, such as the nature of the metal and organic building blocks. The oral lethal dose 50 (LD_50_) of Fe is 30 g/kg, while that of Zn is 350 µg/kg, and that of Zr is 4.1 g/kg [83, 84]. For organic building blocks, the LD_50_ values of terephthalic acid, trimesic acid, 1-methylimidazole and 2-methylimidazole are 5, 8.4, 1.13 and 1.4 g/kg, respectively. In addition, the hydrophobic–hydrophilic balance is also an important parameter [83]. When designing nMOFs, the dosage of metal ions and organic ligands needs to be considered. In addition to these organic compounds, endogenous biomolecules such as nucleotides and phospholipids can be used as ligands to reduce the risk of toxicity [35, 85]. Most safety studies of nMOFs have examined cell lines in vitro. Thus, studies of nMOF absorption, distribution, metabolism and elimination in vivo are urgently needed.

Another challenge to the clinical application of nMOFs is inadequate stability or rapid degradation. Many studies on the stabilities of nMOFs have been conducted in water, but stability under simulated physiological conditions has been less pursued. For example, MIL-101 (Fe) possesses poor stability in phosphate buffer, while MIL-100 (Fe) is stable in water but decomposes after several days [86]. Recently, Cheng et al. [30] coated extracellular vesicles on the surface of nanosized ZIF-8 and found that biomineralized nMOFs retained their nanostructure in PBS (pH = 7.4) but degraded in acidic buffer (pH = 5.0). Decomposition of nMOFs at a desired region is needed for responsive drug release. Differences in degradability can be achieved by selecting different metal ions, organic linkers, and crystalline structures. Thus, the degradation time could be adjusted from a few hours to several weeks [87, 88]. However, the degradation mechanisms of nMOFs in vitro and in vivo require further study.
